# Estimation of children’s thyroid equivalent doses in 16 municipalities after the Fukushima Daiichi Nuclear Power Station accident

**DOI:** 10.1093/jrr/rrac058

**Published:** 2022-09-16

**Authors:** Gen Suzuki, Tetsuo Ishikawa, Takashi Ohba, Arifumi Hasegawa, Haruyasu Nagai, Hirokazu Miyatake, Nobuaki Yoshizawa

**Affiliations:** International University of Health and Welfare Clinic, Tochigi 324-8501, Japan; Radiation Medical Science Centre for the Fukushima Health Management Survey, Fukushima Medical University, Fukushima 960-1247, Japan; Department of Radiation Physics and Chemistry, School of Medicine, Fukushima Medical University, Fukushima 960-1247, Japan; Department of Radiation Health Management, School of Medicine, Fukushima Medical University, Fukushima 960-1247, Japan; Department of Radiation Disaster Medicine, School of Medicine, Fukushima Medical University, Fukushima 960-1247, Japan; Nuclear Science and Engineering Center, Japan Atomic Energy Agency, Ibaraki 319-1195, Japan; Societal Safety and Industrial Innovation Division, Mitsubishi Research Institute, Inc., Tokyo 100-8141, Japan; Societal Safety and Industrial Innovation Division, Mitsubishi Research Institute, Inc., Tokyo 100-8141, Japan

**Keywords:** thyroid equivalent dose (TED), Fukushima, nuclear accident, atmospheric transport, diffusion, and deposition model (ATDM), whereabouts questionnaire

## Abstract

To elucidate the association between radiation dose and thyroid cancer after the 2011 Fukushima Daiichi Nuclear Power Station (FDNPS) accident, it is essential to estimate individual thyroid equivalent doses (TEDs) to children. In a previous study, we reported a methodology for reconstructing TEDs from inhalation. That methodology was based on individual behavioral survey sheets of the Fukushima Health Management Survey (FHMS) combined with a spatiotemporal radionuclides database constructed by an atmospheric transport, diffusion, and deposition model (ATDM)—the Worldwide version of System for Prediction of Environmental Emergency Dose Information (WSPEEDI) in seven municipalities. In the present study, we further refined our methodology and estimated the combined TEDs from inhalation and ingestion among children in 16 municipalities around the nuclear power station utilizing 3256 individual whereabouts questionnaire survey sheets. Distributions of estimated TEDs were similar to estimates based on direct thyroid measurements in 1080 children in Iwaki City, Kawamata Town, Iitate Village, and Minamisoma City. Mean TEDs in 1-year-old children ranged from 1.3 mSv in Date City to 14.9 mSv in Odaka Ward in Minamisoma City, and the 95th percentiles varied from 2.3 mSv in Date City to 28.8 mSv in Namie Town. In the future, this methodology can be useful for the epidemiological studies of thyroid cancer after the FDNPS accident.

## INTRODUCTION

After the Fukushima Daiichi Nuclear Power Station (FDNPS) accident in March 2011, the United Nations Scientific Committee on the Effects of Atomic Radiation (UNSCEAR) estimated that 100 to 500 PBq of radioactive iodine-131, ^131^I, was released from the FDNPS [1]. It is commonly known that many thyroid cancers (more than 4000) were diagnosed among children who consumed ^131^I-contaminated milk after the Chernobyl nuclear power station accident in 1986 [2], so there has been concern about radiation-induced thyroid cancer among children who lived in Fukushima at the time of FDNPS accident. Thus, the Thyroid Ultrasound Examination (TUE) campaign of the Fukushima Health Management Survey (FHMS) began in October 2011, and about 300 000 children have been screened for thyroid abnormalities every 2–3 year by using high-resolution ultrasound equipment [3]. As of June 2021, 265 thyroid cancers (including suspected cases) have been diagnosed in the TUE [4], which is much larger than the sporadic occurrence expected from the National Cancer Registry in Japan. In order to elucidate the causal relationship between these thyroid cancers and the FDNPS accident, it is essential to estimate individual thyroid doses. However, only 1080 children in Iwaki City, Kawamata Town, and Iitate Village had been screened for ^131^I activities in the thyroid in March 2011 [5], which is too few to allow visualization of the spatial and demographic distribution of thyroid doses in Fukushima.

In a previous report [6], we developed a methodology for estimating individual thyroid equivalent doses (TEDs) from inhaled radionuclides. The method utilized personal whereabouts questionnaire survey sheets in combination with a simulation-based spatiotemporal radionuclides concentration database created by a kind of atmospheric transport, diffusion, and deposition model (ATDM), Worldwide version of System for Prediction of Environmental Emergency Dose Information (WSPEEDI), that utilizes a refined source term developed by the Japanese Atomic Energy Agency (JAEA) [7]. By utilizing the database named WSPEEDI_2019DB [8], which was also adopted in the UNSCEAR 2020 Report [9], we estimated TEDs among children in Minamisoma City and Iitate Village, and we compared the estimates with those based on direct thyroid measurements with an NaI(Tl) survey meter [10] or those based on thyroid measurements with a gamma-spectrometer [11]. Our simulation-based TED estimates were compatible with estimates based on direct thyroid measurements [6], but it was unclear whether our methodology could be applied to children living in other areas, such as Iwaki City or Kawamata Town. Moreover, in the former study we estimated TEDs from only inhaled radionuclides for six municipalities, except for Iitate Village where TEDs from both inhalation and ingestion were estimated [6]. In the present study, combined TEDs from inhalation and ingestion are newly estimated among children living in 16 municipalities including Iwaki City and Kawamata Town, and the estimates are compared with estimates based on direct thyroid measurements.

## MATERIALS AND METHODS

### Data setting and ethical issues

**Fig. 1:**
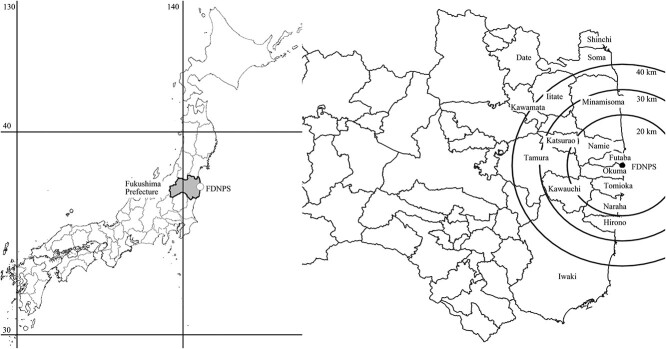
Location of Fukushima Prefecture (left panel) and locations of FDNPS and analyzed 16 municipalities in Fukushima Prefecture (right panel).

After approvals were obtained from the Institutional Review Boards of the International University of Health and Welfare (IUHW: 13-B-185, August 2016; 13-B-339, March 2019) and Fukushima Medical University (FMU: No. 29100, August 2018; No. 2019–003, July 2019), individual questionnaire data of residents who were less than 20 years of age at the time of the accident were randomly selected from the FHMS database and provided to us as anonymized electronic data. We performed the study in accordance with the national ethical guidelines for epidemiological studies and the relevant institutional regulations. According to the guidelines, obtaining new informed consent from a questionnaire provider was not required for the present study. Instead, an opt-out option announced via the WEB pages of FMU and IUHW was deemed to be sufficient. The data set comprised the following items: age, gender, broad location of residence (excluding details of the address, such as house number), locations passed through (including latitude and longitude), length of time spent indoors and outdoors, and travelling time. Questionnaire data from nine municipalities (Iwaki, Soma, Tamura, Date, Hirono, Kawamata, Shinchi, Kawauchi, and Katsurao) were newly obtained in the present study, while data from seven other municipalities (Minamisoma, Iitate, Futaba, Tomioka, Naraha, Okuma, and Namie) had been obtained in the previous study [6]. Table 1 shows numbers of questionnaires obtained from the 16 municipalities and Fig. 1 shows where those municipalities are located.

**Table 1:** Number of whereabouts questionnaire survey sheets obtained from the Basic Survey of Fukushima Health Management Survey

Municipality	Iwaki City	Soma City	Tamura City	Date City	Hirono Town	Kawamata Town	Shinchi Town	Kawauchi Village	Katsurao Village	Minami-soma City	Iitate Village	Futaba Town	Tomioka Town	Naraha Town	Okuma Town	Namie Town
Requested	1000	300	300	300	100	100	100	100	100	300	100	100	100	100	100	100
Received	989	299	297	298	100	100	98	100	100	300	100	100	100	99	100	100
Excluded	9^*^	3^*^	4^*^	2^*^	0	1^*^	2^*^	0	0	1^*^	0	1^*^	0	1^*^	0	0
Number for analysis	980	296	293	296	100	99	96	100	100	299	100	99	100	98	100	100

^^*^^Excluded were individual questionnaire sheets that did not contain complete data for the period 12 to 25 March 2011.

### Estimation of TED from inhalation

A series of Python programs was developed. First, individual questionnaire sheets were resampled so as to make new individual common separated values (CSV) files that contained data on where the respondent was located in coordinates of latitude and longitude, in hourly intervals from 6:00 a.m. on 12 March 2011 to 12:00 p.m. on 25 March 2011. As most people did not specify a route of evacuation but merely described their movement in broad terms, such as ‘from point **A** at time **a** to point **B** at time **b** by car,’ we followed the method adopted in the UNSCEAR 2020 Report [9] and assumed that an evacuee moved in a straight line from point **A** to point **B** at a constant speed to infer hourly location coordinates. Second, for each location coordinate ^131^I concentrations (Bq/m [3]) of three chemical forms (methyl iodide, elemental vapor, and particulate iodine) at a height of 1 m were automatically added to the individual CSV file by referring to the WSPEEDI_2019DB database. Third, the cumulative ^131^I-TED via inhalation from 6:00 a.m. on 12 March to 12:00 p.m. on 25 March 2011 was calculated by formula (1):

(1) \begin{align*} {TED}_{inhal} &= \mathop\sum^{330}_{i} \frac{V}{24} (C_{i-p} \times e_{inhal - p} + C_{i - el} \times e_{inhal - el} \nonumber \\ &\quad + C_{i - met} \times e_{inhal - met} )\times FC\times DF_{shelter} \end{align*}

where *V* is age-specific total daily ventilation volume, *C*_*i-p*,_  *C_i-el_*, and *C_i-met_* are hourly concentrations (Bq/m [3]) of ^131^I-particulate, ^131^I-elemental vapor, and ^131^I-methylated forms, respectively, and *e_inhal-p,_ e_inhal-el,_* and *e_inhal-met,_* are age-dependent TED conversion factors from ICRP publication 71 (Supplementary Table 1A). *FC* is a correction factor for the dose coefficient because the rate of iodine uptake in Japanese is 18.6% (SD 6%), lower than the 30% used in the ICRP thyroid model, whereas thyroid volume in Japanese does not differ from that of the ICRP reference man [12]. *DF_shelter_* is a decontamination factor to reflect sheltering. As reported before [6], we adopted results on *DF_shelter_* reported by Hirouchi *et al.* [13], and *DF_shelter_* after the FDNPS accident in Fukushima Prefecture was estimated by a triangular distribution from 0.1 to 0.95, with a peak at 0.5. As to the decontamination factor for being inside a Japanese car, Iwashita reported that concentrations of particulates of width 2.5 microns (PM2.5) or finer were reduced by more than 0.5 if the vehicle’s internal air circulation mode was selected [14]. As March is still winter season in Fukushima, it is reasonable to think that *DF_shelter_* is applicable to cars during evacuation.

To estimate TED from inhalation of ^131^I and other short-lived radionuclides, i.e. ^132^I, ^132^Te, and ^133^I, ^131^I-TED on 12–13 March 2011 and ^131^I-TED on 15–16 March 2011 were multiplied by age-dependent correction factors, *SF,* as described in the previous study [15]). For 1-year-old children, 1.59 and 1.08 were adopted as *SF* values for plume exposure on 12–13 March and 15–16 March, respectively.

### Estimation of TED from ingestion

Detailed methodology for estimating individual TEDs from ingestion was published elsewhere [16 17]. To apply this methodology to individual Fukushima residents, we had constructed a meta-database of spatiotemporal ^131^I concentrations (Bq/m [3]) in tap water, where hourly ^131^I concentrations from 6 a.m. 12 March to 12 p.m. 25 March 2011 in 3 km grid areas of WSPEEDI_2019DB were calculated with a one-compartment model. For Iwaki City and Iitate Village, ^131^I concentrations in tap water from different water processing facilities were estimated separately. If possible, measured data were used instead of calculated concentrations. Then, a Python program was constructed to estimate TEDs from tap water by utilizing individual CSV files that contained respondents’ hourly location coordinates from 6:00 a.m. on 12 March to 12:00 p.m. on 25 March 2011. TED via ingested tap water was estimated as:

(2) \begin{align*} TED_{ingest} = \mathop\sum_{i}^{330} \frac{pTWI}{24} \times C_{i-tap} \times e_{ingest} \times FC , \end{align*}

where *pTWI* is the age-specific median volume of potentially ingested water per day (Supplementary Table 2) as defined in previous studies^17 18^, *C_i-tap_* is ^131^I concentration in tap water (Bq/m [3]), and *e_ingest_* is an age-dependent TED conversion factor from ICRP Publication 67 (Supplementary Table 1B). We assumed that well water was free of ^131^I contamination; therefore, in the case of Iitate Village, *TED_ingest_* was further reduced by fraction of tap water usage, 0.7, as reported elsewhere [6].

### Uncertainty in TED estimation

To estimate uncertainty of estimated inhalation dose, we assumed the probability density function (PDF) of WSPEEDI to be lognormal with parameters (1 [geometric mean], 3 [geometric standard deviation]) as assumed in the UNSCEAR 2020 Report (Attachment A-12). As to *FC* and *DF_shelter_* in formula (1), we assumed the PDF of *FC* to be normal with parameters (18.6 [mean]) and 6.0 [SD])/30% and that of *DF_shelter_* to be triangular from 0.1 to 0.95 with a peak at 0.5, as reported elsewhere [6]. To estimate the combined uncertainty, a Monte Carlo simulation was repeated 500 000 times using a Latin Hypercube sampling method with the Crystal Ball software (release 11.1.2.3.500, Kozo Keikaku Engineering Inc., Tokyo, Japan). Likewise, to estimate uncertainty of estimated ingestion doses, we assumed the PDF of WSPEEDI estimates to be lognormal as defined above, *pTWI* to be gamma with parameters (θ = 186.15, k = 4.94009) for 1-year-old children, and *FC* to be normal with parameters (18.6, 6.0)/30%. A two-dimensional Monte Carlo simulation was performed 1000 times for the variable part, *pTWI*, with 5000 iterations on the uncertain parts, WSPEEDI and *FC*, using a Latin Hypercube sampling method.

## RESULTS

### Estimated TEDs from inhalation and ingestion among 1-year-old children

In the case of children in evacuation-ordered municipalities, the route and timing of evacuation affected the levels of exposure to radioactive plumes. In the previous study, we reported TEDs from inhalation in each of seven municipalities using ^131^I-concentration in air at 152 representative locations [6]. In the present study, TEDs from inhalation and ingestion were estimated on the basis of ^131^I-concentrations in air at actual locations passed through by the individuals during evacuation. Table 2A shows combined TEDs from inhalation and ingestion of ^131^I, ^132^Te/^132^I, and ^133^I among 1-year-old children in these seven municipalities; the highest estimated mean TED, 14.9 mSv, was among children in Odaka Ward of Minamisoma City, while the highest estimated 95th percentile of TED, 28.8 mSv, was among children in Namie Town.

**Table 2:** Estimated TEDs (mSv) from inhalation and ingestion among 1-year-old children

A									
Municipality	Minami-soma City		Iitate Village	Futaba Town	Tomioka Town	Naraha Town	Okuma Town	Namie Town	
	Odaka Ward	Haramachi/ Kashima wards							
mean	14.9	7.8	8.4	5.7	2.8	3.6	4.6	6.3	
median	15	6	7.6	2.3	1.5	3.3	3.4	2.6	
5th-percentile	0.9	0.6	0.2	0.1	0.0	0.1	0.3	0.2	
25th-percentile	10.8	4.7	2.6	0.8	0.7	0.8	1.5	1	
75th-percentile	19.2	7.8	14.1	4.6	3.4	5.3	5.9	6.6	
95th-percentile	28.4	27	16.6	20.8	10.6	11.1	14.5	28.8	
B									
Municipality	Iwaki City	Soma City	Tamura City	Date City	Hirono Town	Kawamata Town	Shinchi Town	Kawauchi Village	Katsurao Village
mean	6.7	10.4	5.8	1.3	3.3	4.7	10	2.8	2.2
median	5.1	8.3	4.6	1.3	1.8	5.2	11.6	2	0.7
5th-percentile	0.4	1.4	0.4		0.0	0.2	2.0	0.2	0.0
25th-percentile	1.9	4.9	2.5	1.2	0.6	3	5	0.9	0.2
75th-percentile	10.1	16.6	8.9	1.4	3.9	5.8	14	3.6	2.5
95th-percentile	17.2	18.5	13.9	2.3	13.1	9.4	15.9	9.8	11.4

In the present study, nine more municipalities bordering these seven municipalities (Fig. 1) were analyzed. After deliberate evacuation was recommended for people living in areas beyond a 20 km radius and within a 30 km radius from the FDNPS, all children in Katsurao Village and Kawauchi Village were evacuating to the Nakadohri area until 14 March and 16 March 2011, respectively. Likewise, all children in Hirono Town were evacuating until 13 March to either Iwaki City (42%) or other areas (58%), and 7% of children continued to stay in Iwaki City. The estimated mean and 95th percentile of TEDs among 1-year-old children are 2.2 and 2.5 mSv in Katsurao, 2.8 and 3.6 mSv in Kawauchi Village, and 3.3 and 3.9 mSv in Hirono Town (Table 2B).

For Tamura City, the evacuation order was delivered to 7.5% of people living in Miyakoji area, but in fact 55% of children were evacuating until 18 March. Children who did not evacuate tended to receive higher TEDs than those who evacuated. The estimated mean and 95th percentile of TEDs among 1-year-old children are 5.8 and 13.9 mSv, respectively (Table 2B).

As to Shinchi Town, Soma City, and Haramachi and Kashima wards of Minamisoma City located in the northern coastal area (Fig. 1), radioactive plumes drifted over the area on 12, 18, 20 and 22 March [19]. Therefore, children were potentially exposed to multiple plumes, depending on their evacuation scenario. In Shinchi Town, 37.5% of children evacuated before 19 March, but 62.5% of children did not. As to Soma City, 51% of children evacuated before 19 March, but the rest did not. As to Haramachi and Kashima wards of Minamisoma City, 91% of children evacuated as reported in the previous study [6]. As shown in Table 2, the estimated mean and 95th percentile of TEDs among 1-year-old children are 10.0 and 15.9 mSv in Shinchi Town, 10.4 and 18.5 mSv in Soma City, and 7.8 and 27 mSv in Haramachi/Kashima wards (Table 2A, B).

As to Iwaki city, radioactive plumes drifted over the city on 15, 16, 21, and 24 March [19]. Deliberate evacuation was called for, but only 19% of children evacuated before 15 March and 47% children still remained in Iwaki City on 21 March. The estimated mean and 95th percentile of TEDs among 1-year-old children in Iwaki City are 6.7 and 17.2 mSv (Table 2B).

In Kawamata Town located in the north-west direction 30–50 km from the FDNPS (Fig. 1), 18% of children evacuated and the estimated mean and 95th percentile of TEDs are 4.7 and 9.4 mSv. In Date City, located in the north–north-west direction more than 40 km from the FDNPS, only 6% of children evacuated and the mean and 95th percentile of TEDs are 1.3 and 2.3 mSv (Table 2B). Estimated TEDs among 5-, 10-, 15-, and 20-year-old children are shown in Supplementary Table 3.

### Proportions of inhalation- and ingestion-TEDs

**Fig. 2:**
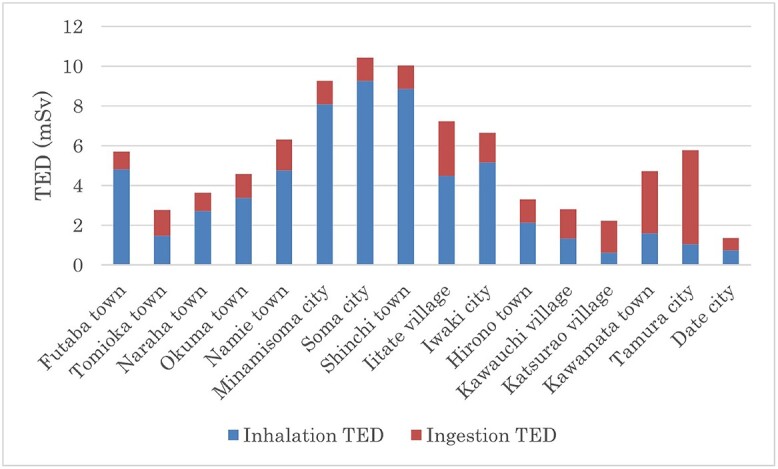
Estimated average thyroid equivalent doses from inhalation and ingestion for 1-year-old children in 16 municipalities. Ingestion TEDs were from tap water usage. Doses from contaminated foods were not estimated in the present analysis.

As shown in Fig. 2, inhalation dose was generally higher than ingestion dose in municipalities that were under evacuation orders and in other coastal municipalities, i.e. Iwaki City, Minamisoma City, Soma City, and Shinchi Town. On the other hand, ingestion dose was relatively higher in inland municipalities, i.e. Iitate Village, Kawauchi Village, Katsurao Village, Kawamata Town, and Tamura City. It should be noted that ingestion dose in Fig. 2 was from contaminated water only. Ingestion dose from foods is discussed later.

### Comparison of simulation-based ^131^I-TED estimates with those based on direct thyroid measurements

To validate estimated ^131^I-TEDs based on individual whereabouts questionnaire sheets in combination with WSPEEDI_2019DB, we compared the estimates with ^131^I-TEDs estimated from direct thyroid measurements made by Kim *et al.* [10] and Tokonami *et al.* [11] Tables 3A and 3B show ^131^I- TED distributions estimated for 5- and 10-year-old children in Iwaki City, Kawamata Town, and Iitate Village. It should be noted that 0 mSv (minimum, 25th percentile, or median values in Table 3) does not necessarily imply no ^131^I activities in the thyroid, but rather reflects difficulty in detecting small amounts of thyroid activities on top of high background counts. Scenarios 1 and 2 in Kim’s report were acute exposure on 15 March 2011 and chronic exposure from 15 March to the day of thyroid measurement; the most probable exposure scenario probably lies between these two [10]. Table 3C shows TEDs estimated for children under 15 years of age at the time of the accident and for adults. As to the scenario 1 for Minamisoma City, we assumed that acute exposure occurred on 12 March instead of 15 March. Our estimated TEDs among 5-year-old children in Iwaki City, Kawamata Town, and Iitate Village are between the figures of scenario 1 and scenario 2 (Table 3A). With the exception of maximum values, our TED estimates among 10-year-old children are greater than the figures of scenario 1 (Table 3B). As to Minamisoma City, our TED estimates are generally in good agreement with estimates based on direct thyroid measurements (Table 3C).

**Table 3:** Comparison of ^131^I-TED (mSv) based on simulation with those based on direct thyroid measurements

A. 5-year-old children									
^131^I-TED centile (mSv)	Iwaki City			Kawamata Town			Iitate Village		
	Kim *et al.* (*n* = 2 V48)		Present study (*n* = 980)	Kim *et al.* (*n* = 333)		present study (*n* = 99)	Kim *et al.* (*n* = 99)		present study (*n* = 100)
	scenario 1^*^	scenario 2^*^^*^		scenario 1^*^	scenario 2^*^^*^		scenario 1^*^	scenario 2^*^^*^	
Minimum	0	0	0	0	0	0	0	0	0
25th-percentile	0	0	1.7	0	0	2.5	0	0	2.3
median	5	2.6	4.6	0	0	4.3	7.3	3	6.4
75th- percentile	10.6	5.2	9.2	5.9	2.7	4.9	14.7	5.9	12.0
Maximum	47.5	25	27.1	29.3	11.9	7.9	29.3	11.9	20.9
B. 10-year-old children									
^131^I-TED centile (mSv)	Iwaki City			Kawamata Town			Iitate Village		
	Kim *et al.* (*n* = 38)		Present study (*n* = 980))	Kim *et al.* (*n* = 156)		present study (*n* = 99)	Kim *et al.* (*n* = 114)		present study (*n* = 100)
	scenario 1^*^	scenario 2^*^^*^		scenario 1^*^	scenario 2^*^^*^		scenario 1^*^	scenario 2^*^^*^	
Minimum	0	0	0	0	0	0	0	0	0
25th-percentile	0.6	0.3	1.4	0	0	2.1	0	0	2.0
median	2.8	1.5	4.0	0	0	3.5	3.7	1.6	5.4
75th- percentile	5.6	2.9	7.8	3.4	1.6	3.9	7.5	3.3	8.9
Maximum	15.3	8.2	23.6	11.2	4.9	6.3	22.4	9.8	17.4
C. TEDs estimated for children under 15 years of age at the time of the accident and for adults									
	Minamisoma City				Minamisoma City, Odaka Ward				
	Kim *et al.* (*n* = 31)		Present study (*n* = 300)		Tokonami *et al.* (*n* = 32)	Present study (*n* = 62)			
	1 ~ 15-year- old children		1 y.0.	15 y.o.	Adult^$^	Adult			
^131^I-TED centile (mSv)	Scenario 1^*^	Scenario 2^*^^*^							
Minimum	0	0	0.4	0.3		0.3			
25th- percentile	0	0	3.3	2.2	1.6	3.4			
median	3.9	1.8	4.6	3	4	4.8			
75th- percentile	10.5	4.6	7.8	5.2	6	6.3			
Maximum	36.8	16	36.4	24.9	44.5	16.1			

^^*^^Scenario 1: acute exposure on 12 March for Minamisoma City and on 15 March for Iwaki City and Kawamata Town.

^^*^^*^^Scenario 2: chronic exposure from 15 March to the time of measurement.

^$^TEDs were estimated under the assumption that residents inhaled on 12 March 2011. One family (five members) was omitted from the analysis as they remained in Namie Town until the end of March.

## DISCUSSION

In the present study, we estimated combined TEDs from inhalation and ingestion among children in 16 municipalities in Fukushima by utilizing 3256 individual whereabouts questionnaire survey sheets, for the first time in combination with WSPEEDI_2019DB. As the proportion of ingestion dose was higher in municipalities located inland (Fig. 2), it is essential to estimate combined inhalation and ingestion TEDs. Estimated TEDs from inhalation and ingestion in children were very close to values estimated from direct thyroid measurements in Iwaki City, Minamisoma City, Iitate Village, and Kawamata Town reported by Kim *et al.* [10] and Tokonami *et al.* [11] Mean TEDs among 1-year-old children in the 16 municipalities ranged from 1.3 mSv in Date City to 14.9 mSv in Odaka Ward in Minamisoma City. The 95th percentile of TEDs ranged from 2.3 mSv in Date City to 28.8 mSv in Namie Town. These estimates are lower than those in the UNSCEAR 2013 Report [1] but are comparable with estimates in the UNSCEAR 2020 Report [9], which utilized the same WSPEEDI 2019DB and 40 representative evacuation scenarios.

The present TED estimates are more sophisticated than those in Ohba’s study [6], where TEDs were estimated by utilizing the same individual whereabouts questionnaire sheets and WSPEEDI_2019DB but with lower resolution: 6-hour averaged ^131^I-concentrations at 152 representative locations in Ohba’s study [6] vs hourly ^131^I-concentrations at locations actually passed through in the present study. In addition, TED via ingestion of tap water is combined on an individual basis in the present study. As the proportion of TEDs via ingestion was higher among residents in inland municipalities, such as Iitate Village, Kawamata Town, or Tamura City (Fig. 2), combined TEDs from inhalation and ingestion are essential for evaluating the spatial distribution of TEDs in Fukushima.

Our simulation-based TED estimates for 5-year-old children are generally in good agreement with those based on direct thyroid measurements (Table 3A), while estimates for 10-year-old children are a bit greater than those based on direct thyroid measurements (Table 3B). If 5- and 10-year-old children had lived in the same environment, age-specific TEDs should not differ by more than 20% (Supplementary Table 3). As ^131^I-activities detected in the thyroid gland were generally higher in 10-year-old-children than in 5-year-old children [10], TEDs for 10-year-old children estimated by Kim *et al.* might be more reliable than those for 5-year-old children. If so, our central estimates could be slightly over-estimated, although they are within the 95% uncertainty intervals (UIs) as discussed below.

It is noted that the proportion of inhalation- and ingestion-TEDs differed among municipalities (Fig. 2): the closer to the FDNPS, the higher the concentration of ^131^I in the air. Thus, people living in evacuation-ordered municipalities near the FDNPS tended to inhale more ^131^I than those in other municipalities. On the other hand, rain and snow precipitated ^131^I from the air onto the ground and contaminated water sources in municipalities located inland, such as Iitate Village, Kawamata Town, or Tamura City, on 15–16 March. Most people in these municipalities stayed and consumed tap water before counter measures were implemented.

### Uncertainty in dose estimation

There are several sources of uncertainty in dose estimation. First, the ATDM simulation may be inaccurate in terms of radionuclide concentrations at particular locations (1 km-grid) and at particular points in time. In the present study, we utilized radionuclide concentrations in a 1 km grid for the ATDM simulation. If radionuclide concentrations in a 3 km grid had been utilized, mean TEDs could have increased by 10% in Namie Town and Minamisoma City and, depending on a child’s place of residence, individual TEDs could be 50% higher or 30% lower than the central estimate (data not shown).

Second, a correction factor for the dose coefficient *FC*, a decontamination factor *DF*_*shelter*,_ and an age-specific potential ingestion volume of tap water per day, *pTWI,* may vary from person to person. To evaluate the combined uncertainty of dose estimation from ATDM, *FC*, *DF_shelter_*, and *pTWI*, Monte Carlo simulation was performed as described in Materials and Methods. As to TED from inhalation in 1-year-old children, the 95% UI ranged from 0.08- to 9.6-fold relative to the central point estimate. As to TED from ingestion in 1-year-old children, the 95% UI ranged from 0.1- to 9.1-fold relative to the central point estimate. Although these 95% UI ranges were large, our assumptions for PDFs in the Materials and Methods section could be conservative, since our central estimates in Table 3 agreed well with analogous estimates based on direct thyroid measurements.

Third, the ratio of ^131^I to ^132^Te or the composition of the three chemical forms of radio-iodine–methyl iodide, elemental iodine, and particulate iodine–might be uncertain. In the present study, we adopted a constant ratio of ^131^I to ^132^Te on 12 March based on the measured values of radionuclides on clothing as reported elsewhere^15 20^, and we estimated TEDs from ^131^I, ^132^Te/^132^I, and ^133^I by utilizing *SF* as described in Materials and Methods. On the other hand, the source term used for the ATDM simulation hypothesized a depletion of ^132^Te in the plume on the afternoon of 12 March [7]. If ^132^Te was actually depleted, we might have overestimated by 20% the inhalation dose due to the 12 March plume among evacuees from Namie Town and Odaka Ward and among residents in the northern coastal area.

Fourth, we assumed that an evacuee moved in a straight line from point A to point B at a constant speed, as mentioned in Materials and Methods. In other words, we assumed a simplified ‘as the crow flies’ route to destination and we ignored variation in speed of movement. This assumption might also be a source of uncertainty in dose estimation.

Fifth, we did not include doses from contaminated food, only those from tap water. Leafy vegetables and milk could be potential sources of ^131^I intake. However, we thought that TEDs from these contaminated foods might be small. Because of disturbances in the milk processing facilities and shipping systems, in addition to transient closure of three major markets in Fukushima and the prolonged closure of many retail stores [21], it was difficult for many residents to consume foods originating from within Fukushima. In addition, monitoring of contaminated vegetables and milk began on 16 March 2011 and restricted distribution began a few days later in Fukushima [21]. Murakami *et al.* estimated TEDs from contaminated water and foods for adults in Fukushima city [22]. In cases where people consumed water and foods bought from retail stores, the TED was estimated to be 0.84 mSv. In cases where people consumed contaminated foods not only bought from retail stores but also those banned from being shipped to retail stores, the TED would be 2.7 mSv. Although some people might have consumed contaminated vegetables grown on private farms or in home gardens, judging from the ^131^I activities in 1080 children [10] or ^137^Cs activities measured by whole body counter [22], such consumption was probably rare.

### Significance of the present study

In the former study, we established a methodology for assessing TEDs based on individual whereabouts questionnaire survey sheets in combination with an ATDM simulation. In the present study, the methodology was further refined to estimate TEDs from combined inhalation and ingestion, and it was validated by comparison with TEDs based on thyroid measurements conducted among 1080 children in Iwaki City, Kawamata Town, Iitate Village, and Minamisoma City. The methodology can be applied for a case–control study to elucidate the relationship between the FDNPS accident and thyroid cancers in Fukushima.

## FUNDING

This work was supported by Research on the Health Effects of Radiation (2014–2016, 2017–2018, 2019–2021) organized by Ministry of the Environment, Japan.

## CONFLICT OF INTEREST

The authors declare they have no conflicts of interest.

## Supplementary Material

Supplementary_Table_1_rrac058

Suplementary_Table_2_rrac058

Supplementary_Table_3_rrac058
