# Supplementary material for: Estimation of children’s thyroid equivalent doses in 16 municipalities after the Fukushima Daiichi Nuclear Power Station accident
Source: J Radiat Res. 2022 Sep 16;63(6):796–804. doi: 10.1093/jrr/rrac058 (PMC9726711; doi:10.1093/jrr/rrac058)
Supplement: Supplementary_Table_1_rrac058 [file supplementary_table_1_rrac058.pdf]

Supplementary Table 1. ICRP age-specific TED conversion factors for TED from inhalation (A) and ingestion (B).

A	Conversion factor			Ventilation rate
	Methyl form (^{131}I)	Elemental vapor form (^{131}I)	Particle aerosol (^{131}I), AMAD=1.0 μm, Type F	
Unit	Sv/Bq			m^3/day
1-year-old	2.5×10^{-6}	3.2×10^{-6}	1.4×10^{-6}	5.16
5-year-old	1.5×10^{-6}	1.9×10^{-6}	7.3×10^{-7}	8.72
10-year-old	7.4×10^{-7}	9.5×10^{-7}	3.7×10^{-7}	15.3
15-year-old	4.8×10^{-7}	6.2×10^{-7}	2.2×10^{-7}	20.1 (male)
Adult	3.1×10^{-7}	3.9×10^{-7}	1.5×10^{-7}	22.2 (male)

-B12:B12:C	Conversion factor	Time after intake
	^{131}I、f1=1.0	
Unit	Sv/Bq	Years
1-year-old	3.6×10^{-6}	69
5-year-old	2.1×10^{-6}	65
10-year-old	1.0×10^{-6}	60
15-year-old	6.8×10^{-7}	55
Adult	4.3×10^{-7}	50
