# Supplementary material for: Estimation of children’s thyroid equivalent doses in 16 municipalities after the Fukushima Daiichi Nuclear Power Station accident
Source: J Radiat Res. 2022 Sep 16;63(6):796–804. doi: 10.1093/jrr/rrac058 (PMC9726711; doi:10.1093/jrr/rrac058)
Supplement: Suplementary_Table_2_rrac058 [file suplementary_table_2_rrac058.pdf]

Supplementary Table 2 Age-specific pTWI (mL/day)

	median	mean	95th-percentile
1-year-old	786	862	1802
5-year-old	1105	1440	2525
10-year-old	1546	1653	2913
15-year-old	1546	1653	2913
adult	1546	1653	2913
