# Supplementary material for: Estimation of children’s thyroid equivalent doses in 16 municipalities after the Fukushima Daiichi Nuclear Power Station accident
Source: J Radiat Res. 2022 Sep 16;63(6):796–804. doi: 10.1093/jrr/rrac058 (PMC9726711; doi:10.1093/jrr/rrac058)
Supplement: Supplementary_Table_3_rrac058 [file supplementary_table_3_rrac058.pdf]

Supplementary Table 3. Estimated TEDs (mSv) from inhalation and ingestion of ^{131}I, ^{132}Te/^{132}I, and ^{133}I for different age categories in 16 municipalities

Tomioka

	1y	5y	10y	15y	20y
mean	2.8	2.4	2.0	1.6	1.1
median	1.5	1.3	1.1	0.9	0.6
5th%-ile	0.0	0.0	0.0	0.0	0.0
25th%-ile	0.7	0.6	0.5	0.4	0.3
75th%-ile	3.4	3.0	2.4	1.8	1.3
95th%-ile	10.6	8.6	7.0	5.7	4.0

Futaba

	1y	5y	10y	15y	20y
mean	5.7	5.2	4.3	3.5	2.5
median	2.3	2.0	1.7	1.3	0.9
5th%-ile	0.1	0.1	0.1	0.1	0.1
25th%-ile	0.8	0.7	0.6	0.5	0.3
75th%-ile	4.6	4.0	3.2	2.5	1.7
95th%-ile	20.8	19.0	16.1	13.4	9.5

Katsurao

	1y	5y	10y	15y	20y
mean	2.2	1.9	1.5	1.1	0.7
median	0.7	0.6	0.5	0.4	0.3
5th%-ile	0.0	0.0	0.0	0.0	0.0
25th%-ile	0.2	0.2	0.1	0.1	0.1
75th%-ile	2.5	2.3	1.9	1.5	1.1
95th%-ile	11.4	9.2	7.2	5.0	3.2

Minamisoma

	1y	5y	10y	15y	20y
mean	9.3	8.4	7.1	5.8	4.1
median	6.5	5.8	4.9	4.1	2.9
5th%-ile	0.6	0.6	0.5	0.4	0.2
25th%-ile	4.9	4.4	3.7	3.1	2.2
75th%-ile	11.2	10.3	8.6	7.0	5.0
95th%-ile	27.2	24.8	21.5	17.8	12.5

Odaka_Minamisoma

	1y	5y	10y	15y	20y
mean	14.9	13.5	11.4	9.4	6.7
median	15.0	13.6	11.5	9.4	6.7
5th%-ile	0.9	0.8	0.7	0.5	0.4
25th%-ile	10.8	9.8	8.3	6.7	4.7
75th%-ile	19.2	17.5	14.8	12.3	8.7
95th%-ile	28.4	25.7	21.6	17.7	12.6

Haramachi_Kashima

	1y	5y	10y	15y	20y
mean	7.8	7.1	6.0	4.9	3.5
median	6.0	5.4	4.5	3.7	2.6
5th%-ile	0.6	0.5	0.4	0.3	0.2
25th%-ile	4.7	4.3	3.6	3.0	2.1
75th%-ile	7.8	6.9	5.8	4.8	3.4
95th%-ile	27.0	24.8	21.5	17.8	12.4

Namie

	1y	5y	10y	15y	20y
mean	6.3	5.7	4.7	3.8	2.7
median	2.6	2.2	1.8	1.4	0.9
5th%-ile	0.2	0.1	0.1	0.1	0.1
25th%-ile	1.0	0.9	0.8	0.6	0.4
75th%-ile	6.6	5.6	4.7	3.9	2.8
95th%-ile	28.8	26.2	22.2	18.4	13.1

Naraha

	1y	5y	10y	15y	20y
mean	3.6	3.4	2.8	2.3	1.6
median	3.3	3.1	2.6	1.9	1.2
5th%-ile	0.1	0.1	0.1	0.1	0.0
25th%-ile	0.8	0.7	0.6	0.5	0.3
75th%-ile	5.3	4.9	4.2	3.4	2.4
95th%-ile	11.1	10.3	8.8	7.1	5.1

Iitate

	1y	5y	10y	15y	20y
mean	8.4	7.6	6.2	4.9	3.3
median	7.6	7.1	5.9	4.8	3.3
5t^{h}%-ile	0.2	0.2	0.2	0.1	0.1
25th%-ile	2.6	2.5	2.1	1.7	1.1
75th%-ile	14.1	12.4	10.2	7.8	5.3
95th%-ile	16.6	14.9	12.1	9.3	6.3

Kawamata

	1y	5y	10y	15y	20y
mean	4.7	4.0	3.2	2.4	1.6
median	5.2	4.4	3.6	2.8	1.9
5th%-ile	0.2	0.2	0.2	0.1	0.1
25th%-ile	3.0	2.6	2.1	1.6	1.1
75th%-ile	5.8	5.0	4.0	3.0	2.0
95th%-ile	9.4	7.7	6.1	4.4	3.0

Okuma

	1y	5y	10y	15y	20y
mean	4.6	4.1	3.4	2.7	1.9
median	3.4	3.1	2.6	2.0	1.4
5th%-ile	0.3	0.2	0.2	0.2	0.1
25th%-ile	1.5	1.4	1.2	0.9	0.6
75th%-ile	5.9	5.3	4.5	3.6	2.4
95th%-ile	14.5	13.0	10.9	8.9	6.3

Soma

	1y	5y	10y	15y	20y
mean	10.4	9.7	8.3	6.8	4.8
median	8.3	7.5	6.4	5.2	3.7
5th%-ile	1.4	1.2	1.0	0.8	0.5
25th%-ile	4.9	4.5	3.8	3.1	2.2
75th%-ile	16.6	15.5	13.4	11.0	7.9
95th%-ile	18.5	17.3	14.9	12.2	8.7

Shinchi

	1y	5y	10y	15y	20y
mean	10.0	9.3	7.9	6.5	4.6
median	11.6	10.8	9.3	7.6	5.4
5th%-ile	2.0	1.8	1.5	1.1	0.8
25th%-ile	5.0	4.5	3.8	3.1	2.2
75th%-ile	14.0	13.0	11.2	9.2	6.6
95th%-ile	15.9	14.8	12.7	10.4	7.4

Tamura

	1y	5y	10y	15y	20y
mean	5.8	4.7	3.8	2.7	1.8
median	4.6	4.0	3.2	2.4	1.6
5th%-ile	0.4	0.4	0.3	0.2	0.2
25th%-ile	2.5	2.2	1.8	1.3	0.9
75th%-ile	8.9	7.2	5.6	4.0	2.6
95th%-ile	13.9	11.1	8.7	6.1	4.0

Date

	1y	5y	10y	15y	20y
mean	1.3	1.2	1.0	0.8	0.5
median	1.3	1.1	1.0	0.8	0.5
5th%-ile	0.7	0.6	0.5	0.4	0.3
25th%-ile	1.2	1.0	0.9	0.7	0.5
75th%-ile	1.4	1.2	1.0	0.8	0.6
95th%-ile	2.3	2.0	1.6	1.2	0.8

Iwaki

	1y	5y	10y	15y	20y
mean	6.7	6.2	5.3	4.3	3.0
median	5.1	4.8	4.1	3.3	2.4
5th%-ile	0.4	0.4	0.3	0.2	0.2
25th%-ile	1.9	1.7	1.5	1.2	0.8
75th%-ile	10.1	9.3	8.0	6.5	4.6
95th%-ile	17.2	15.9	13.6	11.0	7.7

Hirono

	1y	5y	10y	15y	20y
mean	3.3	3.0	2.5	2.0	1.4
median	1.8	1.6	1.3	1.0	0.7
5th%-ile	0.0	0.0	0.0	0.0	0.0
25th%-ile	0.6	0.5	0.5	0.4	0.3
75th%-ile	3.9	3.7	3.2	2.5	1.8
95th%-ile	13.1	11.5	9.9	8.0	5.6

Kawauchi

	1y	5y	10y	15y	20y
mean	2.8	2.5	2.0	1.5	1.1
median	2.0	1.8	1.5	1.2	0.8
5th%-ile	0.2	0.1	0.1	0.1	0.1
25th%-ile	0.9	0.7	0.6	0.5	0.3
75th%-ile	3.6	3.0	2.4	1.9	1.3
95th%-ile	9.8	9.1	7.2	5.0	3.2
